# The effect of pomegranate fresh juice versus pomegranate seed powder on metabolic indices, lipid profile, inflammatory biomarkers, and the histopathology of pancreatic islets of Langerhans in streptozotocin-nicotinamide induced type 2 diabetic Sprague–Dawley rats

**DOI:** 10.1186/s12906-017-1667-6

**Published:** 2017-03-14

**Authors:** Seyedeh Zeinab Taheri Rouhi, Md. Moklesur Rahman Sarker, Asmah Rahmat, Saad Ahmed Alkahtani, Fauziah Othman

**Affiliations:** 1 Department of Nutrition & Dietetics, Faculty of Medicine and Health Sciences, University Putra Malaysia, 43400 Serdang, Selangor Malaysia; 2 Department of Pharmacology, Faculty of Pharmacy, Lincoln University College, No. 2, Jalan Stadium SS 7/15, Kalana Jaya, 47301 Petaling Jaya, Selangor Malaysia; 3 Department of Pharmacy, State University of Bangladesh, 77 Satmasjid Road, Dhanmondi, Dhaka, 1205 Bangladesh; 4 Department of Pharmacolgy, College of Pharmacy, Najran University, Najran, Kingdom of Saudi Arabia; 5 Department of Human Anatomy, Faculty of Medicine & Health Sciences, University Putra Malaysia, 43400 Serdang, Selangor Malaysia

**Keywords:** Diabetes mellitus, Pomegranate juice, Pomegranate seed powder, Anti-oxidant, Anti-inflammatory, Anti-hyperlipidemic, Plasma glucose, Pancreatic health

## Abstract

**Background:**

Type 2 diabetes mellitus (T2DM) is associated with hyperglycemia, inflammatory disorders and abnormal lipid profiles. Several functional foods have therapeutic potential to treat chronic diseases including diabetes. The therapeutic potential of pomegranate has been stated by multitudinous scientists. The present study aimed to evaluate the effects of pomegranate juice and seed powder on the levels of plasma glucose and insulin, inflammatory biomarkers, lipid profiles, and health of the pancreatic islets of Langerhans in streptozotocin (STZ)-nicotinamide (NAD) induced T2DM Sprague Dawley (SD) rats.

**Methods:**

Forty healthy male SD rats were induced to diabetes with a single dose intra-peritoneal administration of STZ (60 mg/kg b.w.) - NAD (120 mg/kg b.w.). Diabetic rats were orally administered with 1 mL of pomegranate fresh juice (PJ) or 100 mg pomegranate seed powder in 1 mL distilled water (PS), or 5 mg/kg b.w. of glibenclamide every day for 21 days. Rats in all groups were sacrificed on day 22. The obtained data was analyzed by SPSS software (v: 22) using One-way analysis of variance (ANOVA).

**Results:**

The results showed that PJ and PS treatment had slight but non-significant reduction of plasma glucose concentration, and no impact on plasma insulin compared to diabetic control (DC) group. PJ lowered the plasma total cholesterol (TC) and triglyceride (TG) significantly, and low-density lipoproteins (LDL) non-significantly compared to DC group. In contrast, PS treatment significantly raised plasma TC, LDL, and high-density lipoproteins (HDL) levels compared to the DC rats. Moreover, the administration of PJ and PS significantly reduced the levels of plasma inflammatory biomarkers, which were actively raised in diabetic rats. Only PJ treated group showed significant repairment and restoration signs in islets of Langerhans. Besides, PJ possessed preventative impact against 2,2-diphenyl-1-picrylhydrazyl (DPPH) radicals almost 2.5 folds more than PS.

**Conclusions:**

Our findings suggest that active constituents with high antioxidant properties present in PJ are responsible for its anti-hyperlipidemic and anti-inflammatory effects, likewise the restoration effect on the damaged islets of Langerhans in experimental rats. Hence, the pharmacological, biochemical, and histopathological profiles of PJ treated rats obviously indicated its helpful effects in amelioration of diabetes-associated complications.

## Background

Type 2 Diabetes Mellitus (T2DM) is known as a vast puzzling health issue in recent century. It is a novel pandemic, which is the fourth or fifth foremost reasons of death in the richest countries, and there is a considerable indication that it is altering into an epidemic in many poor and middle-income societies. T2DM may expose a disorder of the innate immune system in charge of a progressing cytokine-mediated acute phase response. Moreover, hypertension and irregularities of lipoprotein metabolism are usually found in individuals with diabetes [1].

Oxidative stress has a great effect in the beginning and middle of diabetes progress, as well as its related complications. Since oxidative stress owing to generation of free radicals is assumed as the main reason of hyperglycemia, anti-oxidants play potential role for diabetes treatment [2]. Clinical trials and epidemiological studies have established an inverse correlation between intake of fruits and vegetables and the occurrence of diseases such as diabetes, inflammation, cardiovascular disease, cancer and aging- related disorders. The proposed mechanisms and advantageous health effects by phytochemicals existing in vegetables and fruits are due to their anti-oxidation properties [3]. In the other words, oxidative stress could be prevented by dietary intake of antioxidants which could delay and inhibit the oxidation of susceptible cellular substrates. The high phytochemical content and other advantageous micronutrients in fresh fruits and vegetables has been ascribed to their role of human health improvement [4]. Moreover, various in vitro studies in cultured cells have showed that polyphenols may augment glucose uptake by periphery tissues which would decrease glycaemia [5]. The working ways comprise prohibition of gluconeogenesis [6], adrenergic incitement of glucose uptake [7], and motivation of insulin release by pancreatic β-cells [8].

Now-a-days, scientific community has considerable attention to the medicinal properties of functional foods and nutraceuticals for the management of different diseases and improvement of health conditions, such as diabetes, cancer, immunity, prevent oxidation, etc. [9–13]. In traditional medicine, pomegranate (*Punica granatumL.*) is used for the treatment of various sicknesses because of its bioactive compounds. Pomegranate was reported to have antimicrobial, anti-inflammatory, anti-tumor, anti-hepatotoxic, antiviral, anti-diabetic activities, and they can improve oral, skin, and cardiovascular health. Biological actions of pomegranate fruit that lead it to be considered as a healthy fruit [14] are due to its potent phytochemicals contents that scavenge wide spectrum of free radicals [15].

An analysis of the literature shows that duration of phytochemicals extraction and storage conditions may affect bioavailability of these phytochemicals that might be extracted from fruit juices. Hence to avoid this loss, we have used freshly squeezed juice and separated the seeds of pomegranate fruits. In the other hand, although different biological activities of pomegranate have previously been reported, there is no scientific publication evaluating impacts of fresh pomegranate juice in contrast with pomegranate seed powder. Regarding the fact that the majority of researches on hypoglycemic activity of pomegranate seed are concentrated on pomegranate seed oil, they may not be comparable with this work. Specific doses of pomegranate juice and seed have been applied in this study, which makes it different with previous studies; in addition the controversial results in this study might be the most notable part, which make new contributions necessary for future researches. Therefore the phytochemical analysis and antioxidant effect of pomegranate fresh juice and seed powder, as well as their antidiabetic, anti-inflammatory, anti-hyperlipidemic effects, and the health improvement potential of pancreatic islets of Langerhans in streptozotocin-nicotinamide (STZ-NAD) induced diabetic male rats were studied in this work.

## Methods

### Chemicals and reagents

1,1 diphenyl-2-picrylhydrazyl (DPPH), Tris–HCl buffer, and ascorbic acid were purchased from (Sigma- Aldrich (Co. P.O. Box G-0639), St. Louis, MO, USA). Commercial standard pelleted diet was purchased from Lab Diet #5001 (PMI Feeds Inc, St Louis, MO, USA). Streptozotocin (STZ), Nicotinamide (NAD), Glyburide (Glibenclamide), dimethyl sulfoxide (DMSO), DPX Mounting, and Hematoxylin & Eosin were purchased from Sigma-Aldrich Chemical Co. (St. Louis, MO, USA). Ethanol and diethyl ether were purchased from BDH chemicals (Malaysia). Roche/Hitachi cobas C systems commercial kits (Roche Diagnostics GmbH, Mannheim, Germany), and Enzyme Linked Immuno Sorbent Assay (ELISA) kits (CUSABIO BIOTECH CO., LTD., China) were used in this study. The other reagents and chemicals were used of analytical grade.

### Experimental animals

Forty (n = 40) healthy male Sprague Dawley (SD) rats, aged between 10–12 weeks and with body weight of 200–250 g were purchased from the Saintik Enterprise (Malaysia). The rats were conditioned in standard polypropylene cages (four rats/cage) with 12-h light/dark cycles at room temperature (25 ± 2 °C). A commercial standard pelleted diet (Lab Diet No. 500; PMI Feeds Inc, St Louis, MO, USA), with the chemical composition of protein 24%, fat 5.7%, fiber 6.0%, ash 8.0%, and carbohydrate 58%, as well as tap water ad libitum were provided for all the animals. The rats were quarantined for 1 week prior to experiment to acclimatize them to laboratory condition. All the experiments were conducted according to the institutional guidelines for the Animal Care and Use Committee (ACUC) of University Putra Malaysia. An ethical approval of the experimental protocol was obtained from the Animal Care and Use Committee (ACUC) of the University Putra Malaysia (Animal ethics approval number: UPM/FPSK/PADS/BR-UUH/00502).

### Induction of type 2 diabetes

The rats were fasted 12 h before the streptozotocin- nicotinamide injection. A single dose of NAD, solved in normal saline, was administered (120 mg/kg b.w) intraperitoneally. Fifteen minutes after the administration of NAD, a single dose of STZ (60 mg/kg b.w), freshly dissolved in 0.05 M citrate buffer (PH 4.5), was injected through intraperitoneal (i.p) route. Both Streptozotocin (STZ) and Nicotinamide (NAD) were purchased from Sigma-Aldrich Chemical Co. (St. Louis, MO, USA). The development of hyperglycemia was affirmed by the increased fasting blood sugar (FBS), blood taken from tail vein, determined at 72 h and then on day seven after injection [16]. Overnight fasting rats with threshold value of FBS level more than 126 mg/dl or 7 mmol/L was taken as diabetic [17]. Blood glucose measurement was performed with an Accu-Check glucometer (Roche, Germany).

### Preparation of pomegranate juice and extract of seeds powder

Fresh ripe sweet red pomegranate fruits, imported from Spain, were washed, and manually peeled, and the arils were crushed and squeezed with a commercial juicer-blender (National, Japan). The juice was filtered with a Buchner funnel [18] to remove water insoluble materials and stored at −18 ° C. The seeds from the juice preparation were freeze dried at −20 °C separately and ground into powder and stored at −18 °C. In every treatment, 100 mg of powder was diluted with 1 mL distilled water (D.W.) and thoroughly mixed well by vortex [19]. The mixture was then centrifuged at 3000 rpm for 20 min at 4 °C. The collected approximately 1 mL supernatant of the extract was force-fed to each rat of the seed treatment group.

### Preparation of glibenclamide as a standard antidiabetic drug

Glibenclamide (glyburide; Sigma–Aldrich, St. Louis, MO, USA) as a standard drug, was dissolved in DMSO (dimethyl sulfoxide; Sigma–Aldrich, St. Louis, MO, USA) before administered to the rats as an aqueous suspension at a dose of 5 mg/kg body weight [20].

### Experimental design

**Fig. 1:**
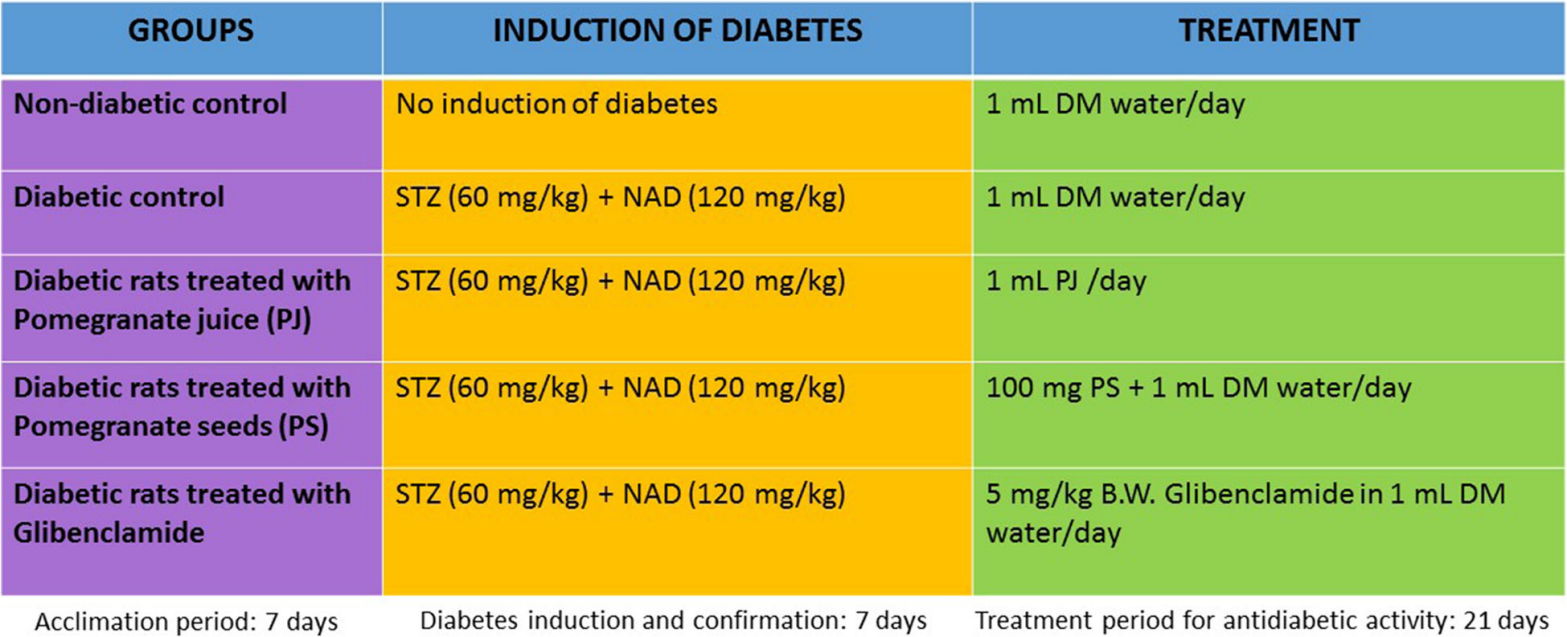
Experimental design. The flowchart shows the research model, grouping of animals, diabetes induction model, type and quantity of different treatments, and time durations in this study

The rats were randomly divided into five groups (*n* = 8) (Fig. 1). Group 1 was normal control rats (NC), group 2: diabetic control (DC), group 3: diabetic rats treated with 1 mL pomegranate juice (PJ), group 4: diabetic rats treated with 100 mg pomegranate seeds powder in 1 mL D.W (PS), and group 5: treated with glibenclamide (5 mg/kg body weight) (G) per day [20]. The rats were treated for 21 days with the oral administration of the PJ, PS, and standard antidiabetic drug, glibenclamide. Rats in all groups were sacrificed on day 22 of treatment [21]. On day 22, blood samples were collected from the overnight (12 h) fasted rats [22] via right atrium (intra-cardiac puncture) after anaesthesized by diethyl ether. The rats were then sacrificed to collect the pancreas for histopathological observation. The plasma samples were obtained through centrifugation the heparinized samples (Rotofix 32, Zentrifugen, Germany) at 4 °C, 3000 rotations per minute (rpm) for 15 min, and transferred into micro centrifuge tubes, using appropriate pipettes. Plasma samples were stored in the −80 °C freezer before further analysis.

### Measurement of body weight

Body weight measures were taken weekly for each animal in all groups by using a digital scale (Sartorius, USA).

### Measurement of plasma glucose and insulin levels

In vivo diagnostic tests for the quantitative determination of the plasma samples in K2 -EDTA tubes were performed to analyze the glucose and lipid profile concentrations in rat plasma according to the methodology proposed by Roche/Hitachi cobas C systems commercial kits (Roche Diagnostics GmbH, Mannheim, Germany) with minor differences in test principles; Roche/Hitachi cobas C systems spontaneously compute the analyte concentration of each sample. Determination of plasma level of glucose, was performed by UV test Enzymatic reference method with hexokinase [23]. Plasma insulin level was defined by rat insulin Enzyme Linked Immunosorbent Assay (ELISA) kit (catalog number: 10-1250-01), purchased from Mercodia AB (Sylveniusgatan 8A, Sweden), and principled as a solid phase two-site enzyme immunoassay method [24].

### Determination of plasma lipid profile

Total cholesterol (TC) and triglyceride (TG) were determined by enzymatic, colorimetric method, and low-density lipoproteins (LDL) and high-density lipoproteins (HDL) were qualified enzymatically with homogeneous enzymatic colorimetric test [25].

### Determination of plasma inflammatory biomarkers

The plasma levels of Interleukin-6 (IL-6), Tumor Necrosis Factor-alpha (TNF-α), and Nuclear Factor-κB (NF-κB) were estimated by the ELISA kits (Cusabio Biotech Co., Ltd., China), with the catalog numbers of CSB-E04640r for the detection of rat IL-6, CSB-E11987r for the detection of TNF-α, and CSB-E13148r for detection of NF-κB were used in this study. In determining all biomarkers, rat standards were prepared in different concentration to determine the accuracy of test, and standard curves were used to determine results after reading the absorbance on spectrophotometer (DYNEX, Technologies MRX II) using 405 nm at primary wavelength and 570 nm as the reference. All samples and standards were assayed in duplicate.

### Histopathological observation of pancreas

At the end of experimental period, all the rat’s abdomens were dissected after being sacrificed; pancreases were immediately excised and trimmed of fatty tissue, washed in saline to remove any red blood cells and clots. Then tail parts (Splenic part) of pancreas were removed and put into 10% neutral buffered formalin for 48 h for histological studies. Tissue processing was done by Autotechnicon and the prepared 5 μ thickness sections were mounted on the microscope slides and stained for the hematoxylin & eosin (H&E) staining of pancreases. The H & E method was applied for quantitative (morphometric) and qualitative (morphologic) analysis. DPX Mounting, and Hematoxylin & Eosin were purchased from Sigma-Aldrich Chemical Co. (St. Louis, MO, USA). The histological features of H&E stained-section of pancreatic tissue were used to investigate the islets of Langerhans damage in rats’ pancreatic tissues. Moreover, average number of Langerhans islets per square centimeter in each section, and average size of Langerhans islets, by measuring diameter of at least four islets in each section and totally 40 islets in each group were assessed [26]. The average number and size of islets of Langerhans in this study have been evaluated by using Cell^ F software (Imaging Software for Life Science Microscopy) via OLYMPUS microscope (Japan).

### Determination of antioxidant activity of pomegranate juice and seed by DPPH radical scavenging assay

Radical scavenging activities of pomegranate juice and seed powders were determined by DPPH radical scavenging assay with some modifications [27]. Briefly, sample stock solutions (5.0 mg/mL) were diluted to final concentrations of 50, 100, 150, 200 and 250 μg/mL in D.W (for PJ) and in ethanol (for seed powder) as the powder was not soluble in D.W. One (1) mL of 0.1 mM DPPH ethanol solution was added to 500 μL of sample solutions of different concentrations. Then 450 μL of 50 mM Tris–HCl buffer (pH 7.4) were added to the mixture. The mixtures were combined well and incubated in the room temperature for 30 min. The reduction of DPPH absorption was measured at 517 nm using a plate reader (BioTek Instruments, Winooski, VT, USA). Ascorbic acid was used as the reference standard. All determinations were performed in triplicate. The Efficient Concentration (EC_50_) value (otherwise called the IC_50_ value) were calculated by linear regression of plots where the abscissa represented the concentration of tested plant extracts, and the ordinate showed the average percent of antioxidant activity. The DPPH radical scavenging activity was calculated using the following equation:

$$ \mathrm{Percentage}\ \mathrm{of}\ \mathrm{inhibition}\ \left(\%\right)=\frac{\mathrm{Absorbance}\ \mathrm{of}\ \mathrm{control}\hbox{-} \mathrm{Absorbance}\ \mathrm{of}\ \mathrm{sample}}{\mathrm{Absorbance}\ \mathrm{of}\ \mathrm{control}}\times 100 $$

### Identification of the bioactive compounds in pomegranate juice and seed by LC-MS/MS (liquid chromatography tandem mass spectrometry)

Liquid chromatography - tandem mass spectrometry (LC-MS/MS) is a sensitive, accurate and specific method coupling high performance liquid chromatography (HPLC) and tandem. Mass Spectrometry (MS-MS) was developed for the separation and identification of phenolic acids in PJ and PS extracts. The pomegranate seed and juice crude samples were extracted with app solvent, and diluted in water to the concentration of 1 mg/ml. Following the dilution, 1 mL of the PS and PJ sample extracts were filtered with nylon 0.22 uM hydrophobic PTFE filter into an auto sampler vial for LC-MS analysis. An AB Sciex 3200QTrap LC-MS/MS with Perkin Elmer FX 15 uHPLC system was used to separate compounds from the pomegranate samples.

### Statistical analysis

The statistical analysis of the data was carried out through SPSS software for Windows (IBM SPSS version 20.0). The statistical significant of data analysis has been assessed by One-way analysis of variance (ANOVA) for the comparison between control group and treatment and within the treatment groups. Significant difference among treatment groups were evaluated by the least significant difference (LSD) range test. The data was reported as the mean ± S.E.M. (standard error of the mean). *P* values < 0.05 considered as statistically significant.

## Results

### Pomegranate juice and seeds extracts could not change the mean body weight of treated diabetic SD rats

Treatment of type 2 diabetic SD-rats with PJ and PS extracts for 21 days could not significantly alter the body weight due to treatment (Table 1). The body weight data showed that there were no significant differences of body weights among the groups, but at the end of the experiment all the control and treated groups had significant weight gain compared to their baseline at week 0 (*P* < 0.05).

**Table 1:** Effect of PJ and PS extracts on body weight gain of experimental diabetic SD rats

Groups	Body weight gain (g)
Normal Control	192.94 ± 11.03
Diabetic Control	101.13 ± 33.13^**^
Pomegranate Seed	77.35 ± 17.43^ns^
Pomegranate Juice	115.88 ± 20.70^ns^
Glibenclamide	102.78 ± 16.33^ns^

SD rats were orally administered with PJ and PS at the doses mentioned earlier for 21 days. The body weights of all rats were measured weekly. The values are means ± S.E.M. of body weight gain for 8 rats in each group. **p˂0.01 was considered as significant compared to nondiabetic control group, *ns* non-significant changes between diabetic treated groups versus diabetic control group after 21 days of treatment

### Pomegranate juice and seed extract non-significantly alter the plasma glucose and insulin levels in treated diabetic SD rats

**Fig. 2:**
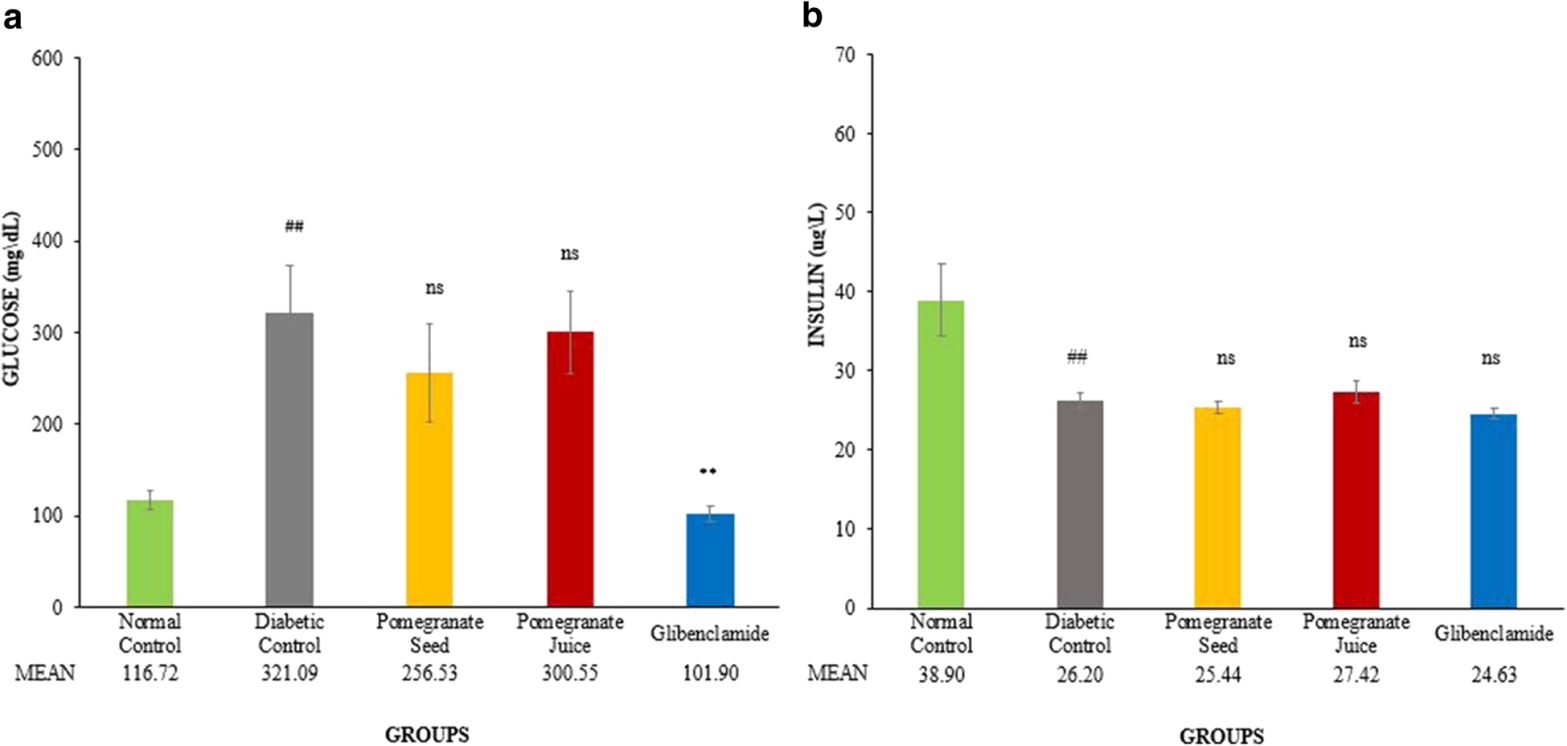
Effect of PJ and PS on plasma glucose and insulin levels of experimental rats. SD rats were orally administered with PJ and PS at the doses mentioned earlier for 21 days. The collected blood from the overnight fasting rats were analyzed for the measurement of plasma glucose **a**, and insulin **b**. The values are means ± S.E.M. of 3 samples/experiments for 8 rats in each group. *p˂0.05, **p˂0.01 were considered significant compared to DC; # *p*˂0.05, ## *p*˂0.01 considered as significant as compared with the normal control. ns: non-significant changes between diabetic treated groups vs. DC group after 21 days treatment

Although the mean level of plasma glucose concentration in diabetic rats receiving PS, and PJ slightly decreased when compared to DC group, none of these changes was statistically significant (*P* > 0.05) (Fig. 2a). The mean level of insulin in DC group decreased significantly (*P* < 0.05) comparing to normal control group. However, none of the treatments significantly affected fasting plasma insulin level in diabetic rats (Fig. 2b). Only glibenclamide significantly reduced fasting plasma glucose, but could not alter the plasma insulin level.

### Plasma bad cholesterols reduced by pomegranate juice, but enhanced by seeds extract

**Fig. 3:**
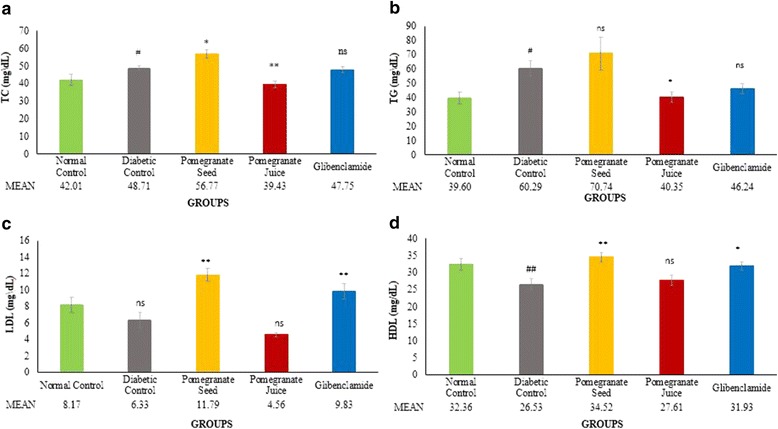
Effect of PJ and PS on plasma lipid profile levels of experimental rats. SD rats were orally administered with PJ and PS at the doses mentioned earlier for 21 days. The collected blood from the overnight fasting rats were analyzed for the measurement of plasma TC **a**, TG **b**, LDL **c**, and HDL **d**. The values are means ± S.E.M. of three samples/experiments for 8 rats in each group.*p˂0.05, **p˂0.01 were considered significant compared to DC; # *p*˂0.05, ## *p*˂0.01 considered as significant as compared with the normal control. ns: non-significant changes between diabetic treated groups vs. DC group after 21 days treatment

As shown in the Fig. 3, the levels of TC and TG were significantly increased, whereas the level of HDL in blood was significantly decreased in diabetic rats compared to non-diabetic rats (# *P* < 0.05, ## *P* < 0.01). Administration of PJ significantly reduced the level of plasma TC (**P < 0.01) and TG (*P < 0.05) (Fig. 3a, b), and non-significantly reduced the level of LDL (Fig. 3c) compared to DC. PJ non-significantly elevated the levels of HDL level (Fig 3d). In contrast, PS treatment significantly raised plasma TC (*P < 0.05) (Fig. 3a) and LDL (**P < 0.01) (Fig. 3c) compared to DC group. Surprisingly, plasma HDL level was also significantly increased by PS (**P < 0.01) (Fig. 3d). PS increased the level of TG as well but the increment level was non-significant (Fig. 3b).

### Anti-inflammatory activities of pomegranate juice and seed extract

**Fig. 4:**
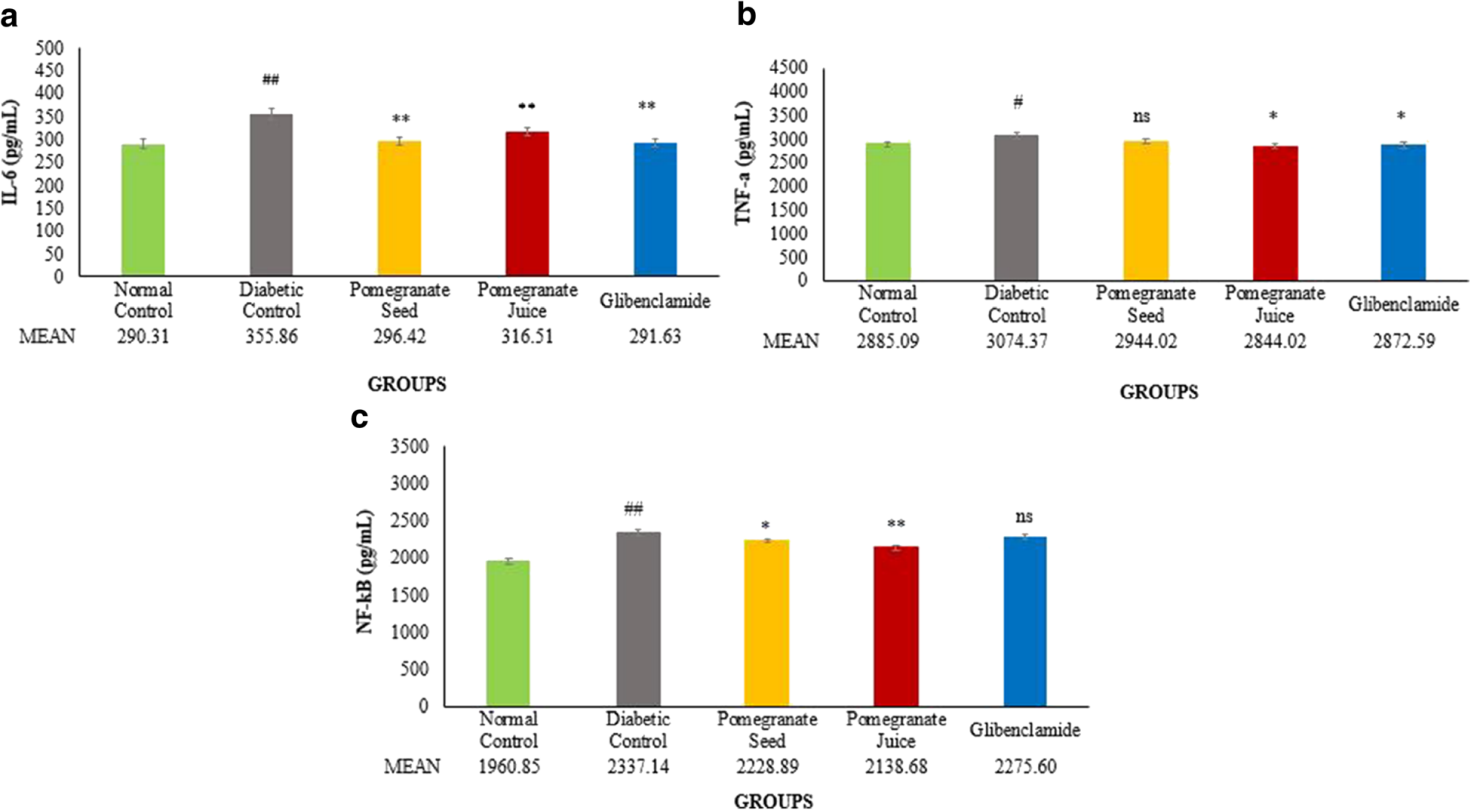
Effect of PJ and PS on plasma pro-inflammatory cytokines levels of experimental rats. SD rats were orally administered with PJ and PS at the doses mentioned earlier for 21 days. The collected blood from the overnight fasting rats were analyzed for the measurement of inflammatory biomarkers: IL-6 **a**, TNF- α **b**, and NF-κB **c**. The values are means ± S.E.M. of three samples/experiments for eight rats in each group. *p˂0.05, **p˂0.01 were considered significant compared to DC; # *p*˂0.05, ## *p*˂0.01 considered as significant as compared with the normal control. ns: non-significant changes between diabetic treated groups vs. DC group after 21 days treatment

There was a significant increase in the concentration of plasma inflammatory biomarkers of IL-6 (## *P* < 0.01), TNF-α (# *P* < 0.05), and NF-κB (## *P* < 0.01) in DC rats compared to normal control group (Fig. 4a, b, and c). Treatment with PJ significantly ameliorated all the tested inflammatory biomarkers: IL-6 (**P < 0.01), TNF-α (*P < 0.05), and NF-κB (**P < 0.01) compared to DC rats group. However, PS treatment also significantly reduced the levels of IL-6 (**P < 0.01) and NF-κB (*P < 0.05) but non-significantly reduced TNF-α level when compared with DC group (Fig. 4a, c, and b, respectively).

### Pomegranate juice improved the number and size of healthy pancreatic islets of Langerhans in diabetic SD rats

**Fig. 5:**
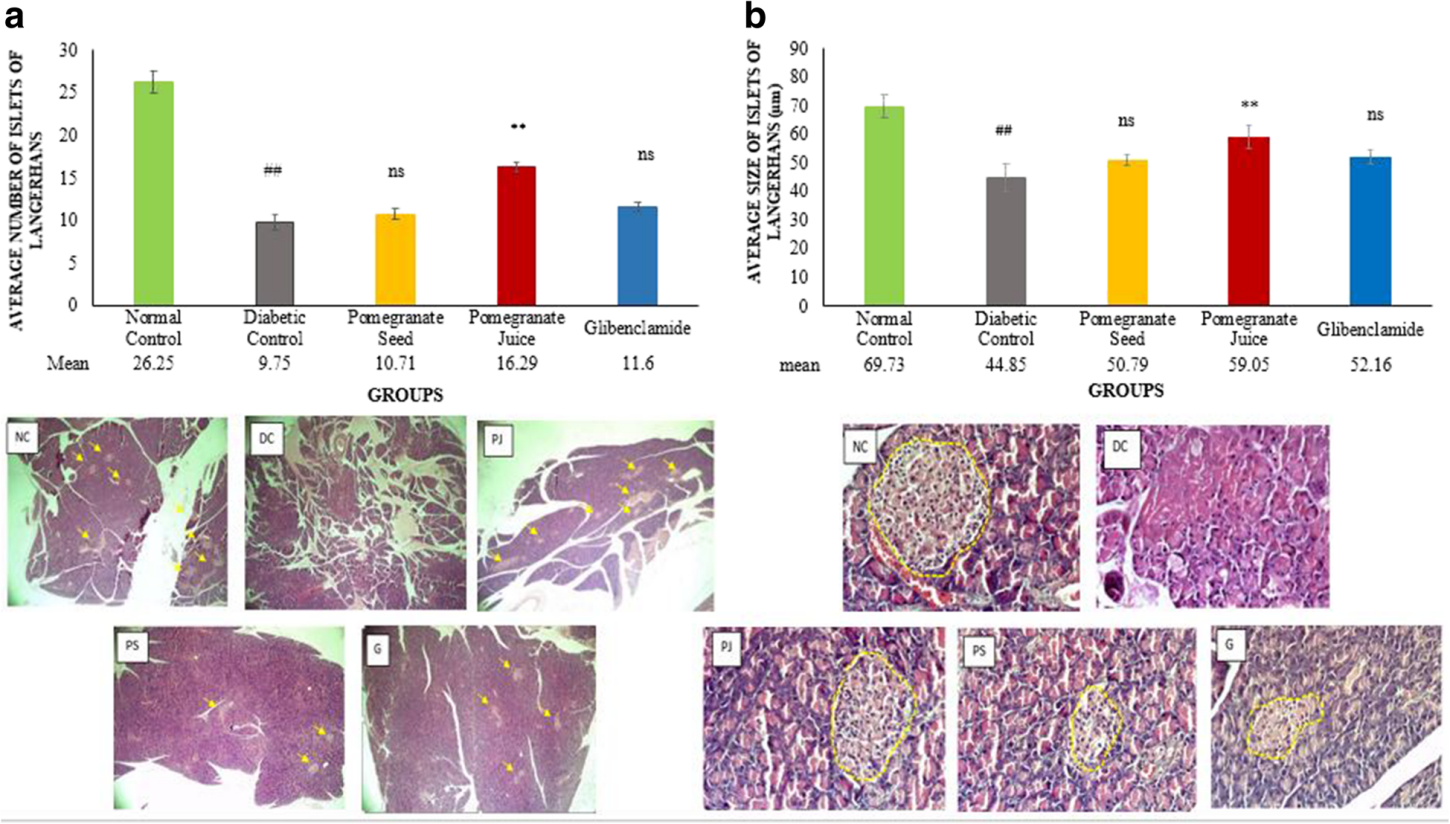
Effect of PJ and PS on histological changes in islets of Langerhans of experimental rats. SD rats were orally administered with PJ and PS at the doses mentioned earlier for 21 days. The collected pancreases from the overnight fasting rats were analyzed for the measurement Average number of islets of Langerhans (per square centimeter) **a** → shows the islets of Langerhans. H & E (×4), and Average size of islets of Langerhans (μm) **b** - - - shows the area of islets of Langerhans. H & E (×40). The values are means ± S.E.M. of three samples/experiments for 8 rats in each group. *p˂0.05, **p˂0.01 were considered significant compared to DC; # *p*˂0.05, ## *p*˂0.01 considered as significant as compared with the normal control. ns: non-significant changes between diabetic treated groups vs. DC group after 21 days treatment

The number and the size of Islets of Langerhans were drastically reduced in DC rats compared with non-diabetic rats (## *P* < 0.01) (Fig. 5a, b). However, treatment of diabetic rats with PJ potentially increased the number of Islets of Langerhans (** *P* < 0.01) compared to DC group (Fig. 5a). Besides, PJ treatment also significantly improved the size of the Islets of Langerhans (** *P* < 0.01) and relative hyperchromic, clear and round nucleus in pancreatic islets were observed in treated group compared to DC group (Fig. 5b). Although pancreases of treated groups with PS and glibenclamide showed slight increment in pancreatic islets, no statistically significant differences were seen in number and size of islets of Langerhans, when compared to DC group. The standard antidiabetic drug used in our study, glibenclamide also could not increase the number and size of islets of Langerhans (Figs. 5a, b).

### DPPH radical scavenging activity of pomegranate juice and seed extracts

**Fig. 6:**
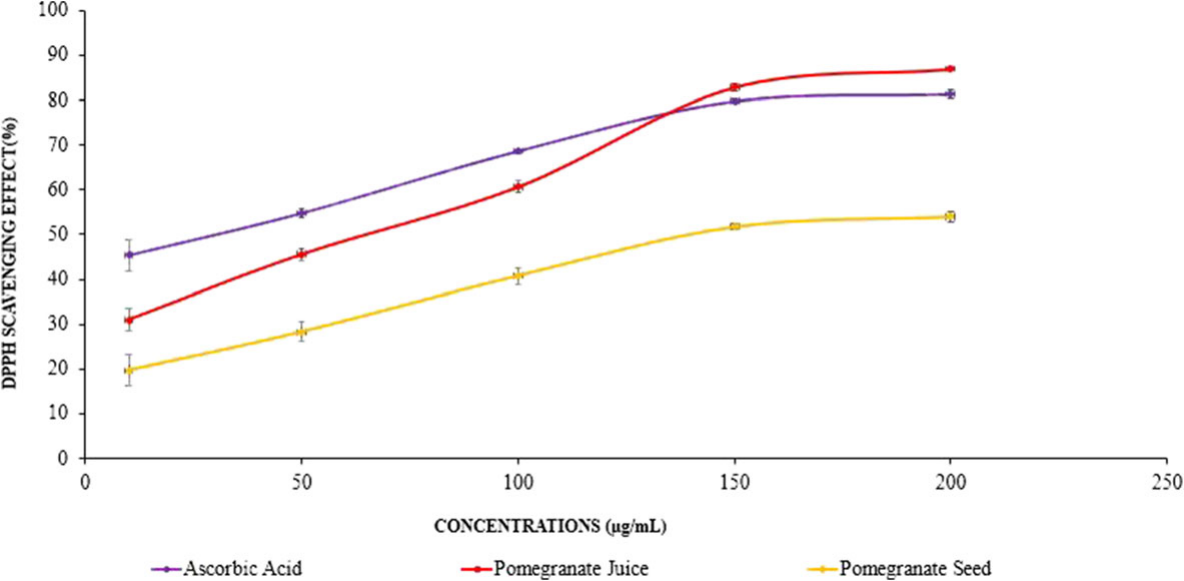
Determination of DPPH inhibition activity of PJ, PS, and ascorbic acid in different concentrations (μg/mL). Antioxidant activity of pomegranate juice, pomegranate seed, and ascorbic acid (as a standard) represented DPPH free radical scavenging effect in a concentration-associated style. The obtained data was the mean of three measurements. The values are means ± S.E.M. of three samples/experiments

The PJ and PS extracts showed significant increase of DPPH free radical scavenging activities in concentration gradient manner. PJ at the concentrations of 150 and 200 μg/mL showed similar DPPH scavenging effect as shown by a standard antioxidant, ascorbic acid (Fig. 6). In all doses (50–200 μg/mL), PJ exhibited much higher free radical scavenging activity compared to PS extract, although pomegranate seed extract significantly increased the free radical scavenging activity. The DPPH scavenging values of PJ, PS, and ascorbic acid at the highest applied dose (200 μg/mL) are 86.89 ± 0.26, 53.98 ± 1.25, and 81.31 ± 0.86%, respectively (Table 2). Considering the IC_50_ values, ascorbic acid showed the lowest IC_50_ value of 21.05 ± 8.05 μg/ml; after that PJ demonstrated the antioxidant activity almost 2.5 folds more than PS; In other words, the PJ could effectively scavenged DPPH (with the IC_50_ value of 65.12 ± 1.44 μg/ml), significantly (*P* < 0.05) stronger than PS (with the IC_50_ value of 159.9 ± 6.24 μg/ml) (Table 2).

**Table 2:** IC_50_ values and DPPH inhibition percentages of sample extracts at the concentration of 200 μg/mL

Samples	Inhibition percentages in concentration 200 μg/mL	IC_50_ values (μg/mL)
Ascorbic acid	81.31 ± 0.86%	21.05 ± 8.05
Pomegranate Juice	86.89 ± 0.26%^**^	65.12 ± 1.44^**^
Pomegranate Seed	53.98 ± 1.25%^***^	159.90 ± 6.24^***^

The values are expressed as means ± S.E.M. for 3 experiments; *p˂0.01, **p˂0.001 are considered as significant compared to standard ascorbic acid

### Phytochemical analysis of pomegranate juice and seed extracts and the possible bioactive compounds

**Fig. 7:**
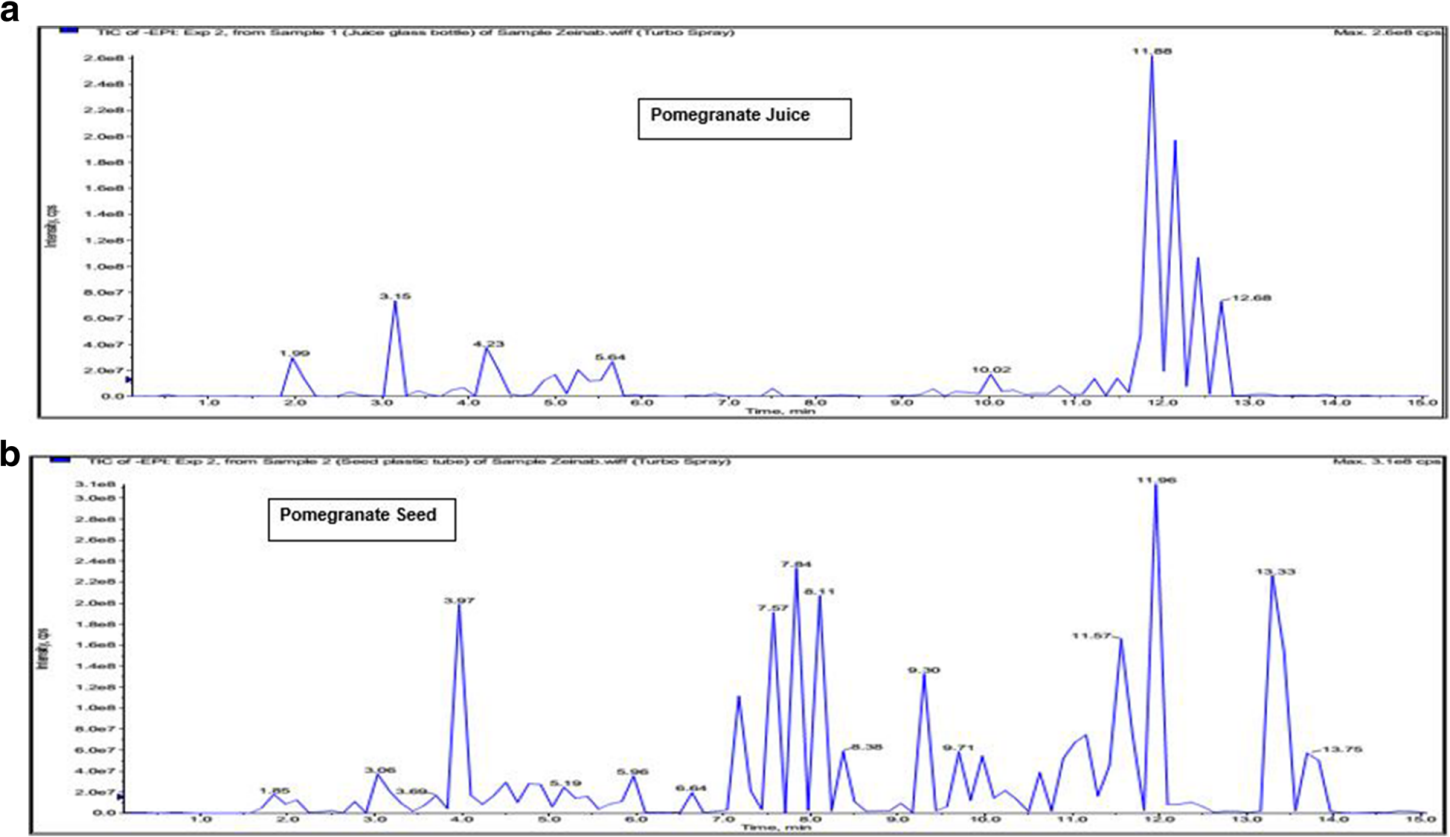
Total ion chromatogram of PJ and PS extracts. The figures show the ion chromatograms of detected compounds in PJ **a** and PS **b** extracts, by using LC-MS/MS analysis

The separation of different compounds in PJ (Fig. 7a) and PS (Fig. 7b) extracts was accomplished by using LC-MS/MS technique in negative mode, which are indicated by the peaks in each graph. LC-MS/MS analysis revealed the presence of ellagic acid in both PJ and PS extracts, as the major compound of PJ, and in smaller amount in PS. It also demonstrated the presence of 3, 30-di-O-methyl ellagic acid, chinic acid, and malic acid in both PJ and PS. It was shown that PJ has catechin, tryptophan, and 15, 16-dihydroxy- 9Z, 12Z-octadecadienoic acid as well. The other compounds found in PS were p-hydroxybenzoic acid, methyl 2-[cyclohex-2-en-1-yl (hydroxy) methyl]-3-hydroxy-4-(2-hydroxyethyl)-3- methyl-5-oxoprolinate, and 3-Oxooctadecanoic acid (stearic acid). Table 3 summarizes the detected compounds in the PJ and PS by LC-MS/MS analysis.

**Table 3:** Compounds detected in pomegranate juice and pomegranate seed extract samples by using LC-MS/MS analysis

Detected compounds	Sample	Description
Chinic acid	Juice/Seed	An acid with anti-inflammatory action
Malic acid	Juice/Seed	The major organic acid in pomegranate
3,30-di-O-methyl ellagic acid	Juice/Seed	An ellagic acid derivative with antioxidant impact
Ellagic acid hexoside	Juice	An ellagic acid derivative with antioxidant impact
Ellagic acid	Juice	A plant derivative polyphenol (the major constituent of PJ with antioxidant impact)
Tryptophan	Juice	An essential amino acid
Catechin	Juice	A polyphenolic component of pomegranate with antioxidant impact
15,16-dihydroxy- 9Z,12Z-octadecadienoic acid	Seed	An unsaturated fatty acid derivative
methyl 2-[cyclohex-2-en-1-yl(hydroxy)methyl]-3-hydroxy-4-(2-hydroxyethyl)-3- methyl-5-oxoprolinate	Seed	An aglycone (an organic compound (as a phenol or alcohol) combined with the sugar portion of a glycoside).
p-hydroxybenzoic acid	Seed	An ester with preservative function in pharmaceuticals, foods, and industry
Oxooctadecanoic acid (stearic acid)	Seed	A saturated fatty acid with an 18 carbon chain (probably the most effective compound on plasma lipid profile)

## Discussion

In the present study, diabetes was induced in experimental SD rats with the administration of nicotinamide and streptozotocin. Streptozotocin is selectively cytotoxic to pancreatic β-cells which damage beta-cells through the production of free radicals and involving with the cellular metabolic oxidative mechanisms [28]. Antioxidants can mitigate oxidative stress produced by STZ induced free radicals by neutralizing the available free radicals. The result of our study showed that both the PJ and PS possess a preventative impact against DPPH free radicals, PJ showed stronger DPPH scavenging activity than PS (Fig 6). The antioxidant activity of PJ is due to the presence of polyphenols ellagic acid and catechins (Table 3). It is assumed that the phytochemical antioxidants including ellagic acid oppose the negative impacts of oxidative stress either by directly working as an antioxidant or by activating/persuading cellular antioxidant enzyme mechanisms [29].

Our results showed a significant augmentation in the levels of three inflammatory biomarkers which are NF-κB, TNF-α, and IL-6, in STZ-NAD induced diabetic rats. However, the levels of those inflammatory cytokines reduced significantly with the treatment of PJ and PS (Fig. 4). It has been observed that decreased insulin sensitivity, decreased glucose tolerance, and increased blood glucose are strongly associated with the two- three fold higher level of inflammatory biomarkers in patients with obesity and type 2 diabetes [30]. Remarkably, treatment with PJ and PS is comparable to glibenclamide in this study, in reduction of IL-6 to the normal. The reason for normalizing the IL-6 in glibenclamide treated animals seems to be the significant lowering of plasma glucose, as well as due to the changes in tissue sensitivity towards insulin. In spite of nonsignificant reduction of plasma glucose levels by PJ and PS, they significantly down regulated the IL-6 levels, most possibly through the scavenging of ROS. PJ treated animals demonstrated a more and statistically significant decrease in TNF-α level compared to PS and glibenclamide treated groups. This significant variation in PJ treated rats might be justifiable with regard to the higher antioxidant activity of PJ, as for ellagic acid, catechins and other compounds in the PJ with the potent antioxidant activity to scavenge reactive oxygen species (ROS). A previous study has shown that ROS causes TNF-α discharge, which influenced with elevated glucose in vitro. Oxidative stress may involve in advancement of a low-grade systemic inflammation in patients with T2DM [31]. NF-κB is a well-known protein that manages the genes encoding the pro-inflammatory cytokines (e. g., IL-1, IL- 2, IL-6, TNF-α, etc.) and some of the acute phase proteins and immune receptors, which have important characters in managing many inflammatory procedures [32]. In the present study, the plasma concentration of NF-κB was significantly increased in diabetic rats, which was then significantly reduced to normal with the treatment of PJ and PS. On the contrary, the standard antidiabetic drug, glibenclamide, could not reduce the level of NF-κB in diabetic rats (Fig. 4c). This betterment in level of inflammation after PJ and PS treatment seems to be because of the presence of bioactive compounds in PJ and PS with inhibitory function on NF-κB pathway. Numerous plant-extracted components has been assessed as probable inhibitors of the NF-κB pathway. These comprise a broad variety of compounds categories including polyphenols [33]. As ROS may also perform to upregulate the expression of pro-inflammatory gene by enabling NF-κB, various agents that lead to oxidative stress can stimulate NF-κB. Some fruits including pomegranate have been illustrated to interface with various pathways implicated in inflammation including NF-κB activation to affect the expression of cytokines, possibly regarding its polyphenol characteristics [34]. The anti-inflammatory impacts and main mechanisms of PJ are due to its ellagic acid and catechin contents.

The lipid profile (TC, TG, LDL, and HDL) was significant elevated in STZ-NAD induced diabetic rats. With the administration of PJ, the levels of plasma TC, TG and LDL were significantly reduced (Fig. 3); whereas, a slight increment in HDL cholesterol in this group was observed which was comparable with DC group. The result of the present study is similar with a previous study in which significant reductions were recorded in LDL and total cholesterol, whereas no difference in HDL was found [35]. Analysis of PJ in this study revealed that PJ is rich in polyphenols and demonstrate high capability in scavenging free radicals and inhibiting LDL oxidation in vitro and in vivo [36]*.* One of the possible actions of PJ may be due to its inhibition of endogenous synthesis of lipids. Enzymes activities propose that enhanced lipid metabolism during diabetes is shifted towards carbohydrate metabolism and it enhances the utilization of glucose at the peripheral sites [37]. It has been stated that polyphenols of PJ also increase the activity of serum HDL associated paraoxonase 1, which can in turn hydrolyze lipid peroxides in oxidized-LDL and convert them to a less atherogenic LDL; thus causing more reduction in oxidized-LDL content [38]. It can be understood from the data that PJ decreases the plasma lipids levels, which are effectively augmented in STZ induced diabetic rats. Moreover, either raised excretion and reduced absorption of cholesterol or a direct effect of flavonoids on cholesterol metabolism or on the activity of hydroxymethyl glutaryl-CoA reductase and sterol O-acyltransferase —two key enzymes in cholesterol metabolism [39] are probably the reasons for the flavonoids’ effect on cholesterol metabolism. It is likely that PJ-induced favorable changes in the lipid profile in diabetic rats may not only be due to better glycemic control, but could also be due to its direct action on lipid metabolic pathways. Therefore, PJ consumption may modify the risk factors regarding the hyperlipidemia in diabetic patients and its inclusion. On the other hand, PS treatment significantly raised plasma TC, LDL, and HDL levels (Fig. 3a, c, and d); plasma TG level also had a non-significant elevation in comparison to DC group (Fig. 3b). According to previous studies, serum TG level should be increased when certain conjugated linolenics are administered orally [40]. It can be the reason of increased TG by PS treatment, compared to DC rats in the present study. Although LC-MS/MS analysis in the present study revealed that PS contains ellagic acid (an antioxidant agents), it did not cause any reduction in lipid profile levels; this is probably due to more stearic acid content of PS treatment applied in this research, in comparison to unsaturated fatty acids. In order to better understanding of these achieved results, it can be helpful to perform the quantity analysis of the discovered components in our pomegranate samples. Further studies are required to elucidate the detailed mechanism of action of PS in vivo, especially with regard to its metabolic effects.

Although PJ and PS could not significantly alter plasma glucose and insulin levels, PJ significantly improved the size of islets of Langerhans, enlarged consequentially as compared with DC rats (Fig. 5). PJ also enhanced the number of islets of Langerhans. The PJ might have some chemical elements that have regenerative impact on pancreatic islets cells and arouse the β-cells to generate more insulin or it may have insulin-like components. PJ, which possessed strong antioxidant property can act as a free radical scavenger and defend pancreatic cells from destruction. This may be regarding the existence of vitamin C and polyphenolic contents such as ellagic acid derivatives and catechins in PJ as potent antioxidants. Nevertheless, PJ treatment did not lead to an increase in the concentration of plasma insulin in this group. The results suggest that augmentation of pancreatic islets with no alteration in insulin level may be due to the improvement of other types of pancreatic cells particularly α-cells, much more than β-cells. Pancreases of PS treated groups showed close similarity to diabetic untreated group. However, the pancreatic damage observed in PS treated diabetic animals was milder than that found in the untreated DC group. Similarly, pancreas of glibenclamide treated rats showed milder damage in comparison to that observed in PS treated groups. PS treatment did not show any considerable changes in the number of islets of Langerhans; only a negligible improvement in islets size was observed compared to DC group. In the other hand, the group treated with PS showed greater degree of necrotic changes as compared to the other treated diabetic rats. Since the improvement in glibenclamide treated rats was not significant, additionally, there was no obvious difference between the mean concentration of insulin in this group versus diabetic rats without treatment, therefore it’s proposed that the probable mechanisms by which glibenclamide brings about its antihyperglycemic activity might not be by pancreatic secretion potentiation of insulin from pancreatic β-cells.

## Conclusion

The present study demonstrated that PJ has antioxidant, anti-inflammatory, lipid lowering activity and to improve the health of pancreatic islets of Langerhans in diabetic SD rats. Therefore, the consumption of PJ would be helpful to control or prevent oxidation, inflammation and bad cholesterols in diabetic patients. The present findings support the traditional usage of PJ for controlling hyperlipidemia in diabetic patients, hence more investigation with longer period or higher dose may show clearer feature of this finding. Therefore, drinking of PJ or utilization of this juice in medicine may help to diabetic patients with lipid abnormalities and related diseases, such as hypercholesterolemia and atherosclerosis. As PS significant increased bad cholesterols, it is recommended to not to consume PS by the individuals suffering from lipoproteins disorders or diseases related to lipid profiles, although it has positive impact on oxidation and inflammatory biomarkers. Although few reports published on the improvement of the glucose and insulin levels, our study resulted no significant changes in the glucose and insulin levels due to PJ treatment in diabetic rats. The exact reason is not known to us. However, further investigations are required to clarify the clear picture on the health benefit of pomegranate for the improvement of oxidative state, to control bad cholesterols, inflammation, and hyperglycemia. Also need to confirm the impact of pomegranate on the improvement of pancreatic beta-cells health. Hence, extensive studies on the pharmacological, biochemical, and histopathological in vitro, in vivo and clinical trials are suggested for further studies.

## Abbreviations

ANOVA: Analysis of variance
DC: Diabetic control
DPPH: 2,2-diphenylpicrylhydrazyl
DPX: Distrene, plasticiser, xylene
D.W: Distilled water
EC_50_: Efficient concentration
EDTA: Ethylene diamine tetraacetic acid
ELISA: Enzyme Linked Immune-Sorbent Assay
FBS: Fasting blood sugar
G: Glibenclamide
H&E: hematoxylin & eosin
HDL: High-density lipoprotein
i.p: Intraperitoneal
IL-6: Interleukin 6
LCMS/MS: Liquid Chromatography-Tandem Mass Spectrometry
LDL: Low-density lipoprotein
LSD: Least significant difference
NC: Normal control
NAD: Nicotinamide
NF-κB: Nuclear Factor kappa-light-chain-enhancer of activated B cells)
PJ: Pomegranate juice
PS: Pomegranate seed
ROS: Reactive oxygen species
S.E.M: Standard error of the mean
STZ: Streptozotocin
T2DM: Type 2 diabetes mellitus
TG: Triglycerides
TNF-α: Tumor necrosis factor α
