# Erratum to: The effect of pomegranate fresh juice versus pomegranate seed powder on metabolic indices, lipid profile, inflammatory biomarkers, and the histopathology of pancreatic islets of Langerhans in streptozotocin-nicotinamide induced type 2 diabetic Sprague–Dawley rats

**DOI:** 10.1186/s12906-017-1724-1

**Published:** 2017-04-13

**Authors:** Seyedeh Zeinab Taheri Rouhi, Md. Moklesur Rahman Sarker, Asmah Rahmat, Saad Ahmed Alkahtani, Fauziah Othman

**Affiliations:** 1 Department of Nutrition & Dietetics, Faculty of Medicine and Health Sciences, University Putra Malaysia, 43400 Serdang, Selangor Malaysia; 2 Department of Pharmacology, Faculty of Pharmacy, Lincoln University College, No. 2, Jalan Stadium SS 7/15, Kalana Jaya, 47301 Petaling Jaya, Selangor Malaysia; 3 Department of Pharmacy, State University of Bangladesh, 77 Satmasjid Road, Dhanmondi, Dhaka, 1205 Bangladesh; 4 Department of Pharmacology, College of Pharmacy, Najran University, Najran, Kingdom of Saudi Arabia; 5 Department of Human Anatomy, Faculty of Medicine & Health Sciences, University Putra Malaysia, 43400 Serdang, Selangor Malaysia

## Erratum

Upon publication of the original article [1], it was noticed that the Family name of the author “Seyedeh Zeinab Taheri Rouhi”, incorrectly appeared as “Rouhi” on the PubMed platform. This was due to incorrect XML Tagging of the authors Family name “Taheri” being marked up as a Given name and has since been updated online.
